# Twelfth degree spline with application to quadrature

**DOI:** 10.1186/s40064-016-3711-2

**Published:** 2016-12-16

**Authors:** P. O. Mohammed, F. K. Hamasalh

**Affiliations:** Department of Mathematics, College of Education, University of Sulaimani, Sulaimani, Kurdistan Region Iraq

**Keywords:** Interpolation, Spline approximation, Quadrature, 65D05, 65D07, 41A15

## Abstract

In this paper existence and uniqueness of twelfth degree spline is proved with application to quadrature. This formula is in the class of splines of degree 12 and continuity order $C^{12}$ that matches the derivatives up to order 6 at the knots of a uniform partition. Some mistakes in the literature are pointed out and corrected. Numerical examples are given to illustrate the applicability and efficiency of the new method.

## Background

In the last two decades, Clarleft et al. (1967) have constructed a direct cubic spline that fits the first derivatives at the knots together with the value of the function and its second derivative at the beginning of the interval. They used it for the solution quadrature formula.

El Tarazi and Karaballi (1990) have constructed five types of even degree splines ($j=2k,\,\,k=1,2,3,4,5$) that match the derivatives up to the order *k* at the knots of a uniform partition for each $k=1,\,2,\,3,\,4$, and 5. These splines are also applied to quadrature.

Recently, Rathod et al. (2010) presented a formulation and study of an interpolatory cubic spline (named Subbotin cubic spline) to compute the integration over curved domains in the Cartesian two space and the integral approximations (quadrature).

In this work, we construct a twelfth degree spline which interpolates the derivatives up to the order 6 of a given function at the knots and its value at the beginning of the interval. We obtain a direct simple formula for the proposed spline. Error bounds for the function is derived in the sense of the Hermite interpolation. Also, a mistakes in the literature was corrected. Finally, numerical examples and comparison with other available methods are presented to illustrate the usefullness of proposed method.

## Description of the spline (existence and uniqueness)

We construct here a class of interpolating splines of degree 12. Error estimates for this spline is also represented.

Let $0=x_{0}<x_{1}<\cdots<x_{n-1}<x_{n}=1$ be a uniform partition of [0, 1]. We denote by $S^{(6)}_{n,12}$ the linear space of twelfth degree spline *s*(*x*) such that

1. $$s(x)\in C^{(6)}[0,1]$$;
2. *s*(*x*) is a polynomial of degree 12 in each subinterval $[x_i,x_{i+1}]$.

Set the stepsize $h=x_{i+1}-x_{i}\,(i=0(1)n)$. Note that if *g* is a real-valued function in [0, 1], then $g_{i}$ stands for $g(x_{i})\,(i=0(1)n)$.

### **Theorem 1**

*Let* *s*(*x*) *be the spline defined in section* “Description of the spline (existence and uniqueness)”. *Given the real numbers* $f_{0}$ *and* $s^{(k)}_{i}=f^{(k)}_{i}$ *for* $\,\,i=0(1)n,\,\,k=1(1)6$. *Then, there exist a unique* $s(x)\in S^{(6)}_{n,12}$ *such that*

1 $$\begin{aligned} {\left\{ \begin{array}{ll} s^{(k)}_{i}&{}=f^{(k)}_{i} \quad i=0(1)n,\,\,k=1(1)6;\\ s_{0}&{}=f_{0} \end{array}\right. } \end{aligned}$$

*The twelfth degree spline* *s*(*x*) *which satisfies* (1) *in* $[x_{i},x_{i+1}]$ *is of the form:*

2 $$\begin{aligned} s(x)=\sum _{j=0}^{11} h^{j}\left[ s^{(j)}_{i}A_{j}(t)+s^{(j)}_{i+1}A_{j}(t)\right] +h^{12}f^{(12)}_{i}A_{12}(t) \end{aligned}$$

*where*

$$\begin{aligned} A_{0}(t)&=(t-1)^6\left( 462t^6+252t^5+126t^4+56t^3+21t^2+6t+1\right) ,\\ A_{1}(t)&=-t^7\left( 462t^5-2520t^4+5544t^3-6160t^2+3465t-792\right) ,\\ A_{2}(t)&=t(t-1)^6\left( 252t^5+126t^4+56t^3+21t^2+6t+1\right) ,\\ A_{3}(t)&=t^7(t-1)\left( 210t^4-924t^3+1540t^2-1115t+330\right) ,\\ A_{4}(t)&=\frac{1}{2}t^2(t-1)^6\left( 126t^4+56t^3+21t^2+6t+1\right) ,\\ A_{5}(t)&=-\frac{1}{2}t^7(t-1)^2\left( 84t^3-280t^2+315t-120\right) ,\\ A_{6}(t)&=\frac{1}{6}t^3(t-1)^6\left( 56t^3+21t^2+6t+1\right) ,\\ A_{7}(t)&=\frac{1}{6}t^7(t-1)^3\left( 28t^2-63t+36\right) ,\quad A_{8}(t)=\frac{1}{24}t^4(t-1)^6\left( 21t^2+6t+1\right) ,\\ A_{9}(t)&=\frac{-1}{24}t^7(t-1)^4(7t-8), \qquad \quad \quad A_{10}(t)=\frac{1}{120}t^5(t-1)^6(6t+1), \\ A_{11}(t)&=\frac{1}{120}t^7(t-1)^5, \qquad \qquad \qquad \quad A_{12}(t)=\frac{1}{720}t^6(t-1)^6, \end{aligned}$$

*and* $x=x_{i}+th$, $t\in [0,1]$, *with a similar expression for* *s*(*x*) *in* $[x_{i-1},x_{i}]$.

*The coefficient* $s_{i}$ in (2) *are given by the* *recurrence formula:*

3 $$\begin{aligned} {\left\{ \begin{array}{ll} s_{i}&{}=s_{i-1}+\frac{1}{2}h\left[ f'_{i-1}+f'_{i}\right] +\frac{5}{44}h^{2}\left[ f''_{i-1}-f''_{i}\right] +\frac{1}{66}h^{3}\left[ f^{(3)}_{i-1}+f^{(3)}_{i}\right] \\ &{}+\frac{1}{792}h^{4}\left[ f^{(4)}_{i-1}-f^{(4)}_{i}\right] +\frac{1}{15840}h^{5}\left[ f^{(5)}_{i-1}+f^{(5)}_{i}\right] +\frac{1}{665280}h^{6}\left[ f^{(6)}_{i-1}-f^{(6)}_{i}\right] \\ s_{0}&{}=f_{0}. \end{array}\right. } \end{aligned}$$

### *Proof*

We can express any polynomial *p*(*t*) in [0, 1] of degree 12 in terms of its values and its derivatives upto order 5 at 0 and 1, and its sixth derivative at 0,

$$\begin{aligned} p(t)=\sum _{j=0}^{5}\left[ p^{(j)}_{0}A_{j}(t)+p^{(j)}_{1}A_{j+1}(t)\right] +p^{(6)}_{0}A_{12}(t) \end{aligned}$$

and to determine the coefficients $A_{j},\,j=0,1,\ldots ,12$, we write the above equality for $p(t)=1,\,t,\,t^{2},\ldots ,t^{12}$, we obtain the following system:

$$\begin{aligned} \begin{array}{cccccccc} A_{0} &{} +A_{1} &{} &{} &{} &{} &{} &{} =1 \\ A_{1} &{} +A_{2} &{} +A_{3} &{} &{} &{} &{} &{} =t \\ A_{1} &{} +2A_{3} &{} +2A_{4} &{} +2A_{5} &{} &{} &{} &{} =t^2 \\ A_{1} &{} +3A_{3} &{} +6A_{5} &{} +6A_{6} &{} +6A_{7} &{} &{} &{} =t^3 \\ A_{1} &{} +4A_{3} &{} +12A_{5} &{} +24A_{7} &{} +24A_{8} &{} +24A_{9} &{} &{} =t^4 \\ A_{1} &{} +5A_{3} &{} +20A_{5} &{} +60A_{7} &{} +120A_{9} &{} +120A_{10} &{} +120A_{11} &{} =t^5 \\ A_{1} &{} +6A_{3} &{} +30A_{5} &{} +120A_{7} &{} +360A_{9} &{} +720A_{11} &{} +720A_{12} &{} =t^6 \\ A_{1} &{} +7A_{3} &{} +42A_{5} &{} +210A_{7} &{} +840A_{9} &{} +2520A_{11} &{} &{} =t^7 \\ A_{1} &{} +8A_{3} &{} +56A_{5} &{} +336A_{7} &{} +1680A_{9} &{} +6720A_{11} &{} &{} =t^8 \\ A_{1} &{} +9A_{3} &{} +72A_{5} &{} +504A_{7} &{} +3024A_{9} &{} +15120A_{11} &{} &{} =t^9 \\ A_{1} &{} +10A_{3} &{} +90A_{5} &{} +720A_{7} &{} +5040A_{9} &{} +30240A_{11} &{} &{} =t^{10} \\ A_{1} &{} +11A_{3} &{} +110A_{5} &{} +990A_{7} &{} +7920A_{9} &{} +55440A_{11} &{} &{} =t^{11} \\ A_{1} &{} +12A_{3} &{} +132A_{5} &{} +1320A_{7} &{} +11880A_{9} &{} +95040A_{11} &{} &{} =t^{12} \end{array} \end{aligned}$$

Solving this system, to obtain $A_{j},\,\,j=0(1)12$, above.

Now for a fixed $i\in \{0,\,1,\ldots ,n\}$, set $x=x_i+th,\,\,0<t<1$. In $[x_i,x_{i+1}]$ the spline *s*(*x*) of degree 12 satisfying (1) is

$$\begin{aligned} s(x)&=\sum _{j=0}^{11} h^{j}\left[ s^{(j)}_{i}A_{j}(t)+s^{(j)}_{i+1}A_{j}(t)\right] +h^{12}f^{(12)}_{i}A_{12}(t) \end{aligned}$$

We have a similar expression for *s*(*x*) in $[x_{i-1},x_i]$. Since $s(x)\in C^{(6)}[0,1]$, we have

$$\begin{aligned} s^{(6)}(x_{i}^{-})&=s^{(6)}(x_{i}^{+}),\quad \hbox {for}\ i=0(1)n,\\ s^{(6)}(x_{n+1}^{-})&=f^{(6)}_{n+1}. \end{aligned}$$

This gives the above recurrence formula (3). Thus, the proof is completed. $\square$

## Error bounds

In this section, error estimates for the above interpolatory twelfth spline is considered. Note that $\Vert \cdot \Vert$ represents the $L_{\infty }$ norm.

### **Theorem 2**

(Birkhoff and Priver 1967; Clarleft et al. 1967; Varma and Howell 1983) *Let* $g\in C^{2m}[0,h]$ *be given. Let* $p_{2m-1}$ *be the unique Hermite interpolation polynomial of degree* $2m-1$ *that matches* *g* *and its first* $m-1$ *derivatives* $g^{(r)}$ *at* 0 *and* *h*. *Then*

4 $$\begin{aligned} \left| e^{(r)}(x)\right| \le \frac{h^{r}[x(h-x)]^{m-r}\,G}{r!(2m-2r)!},\quad r=0(1)m; \quad 0\le x\le h, \end{aligned}$$

*where*

5 $$\begin{aligned} \left| e^{(r)}(x)\right| =\left| g^{(r)}(x)-p^{(r)}_{2m-1}(x) \right| \ \quad \text {and} \quad G=\max _{0\le x\le h}\left| g^{(2m)}(x)\right| . \end{aligned}$$

*The bounds in* (2) *are best possible for* $r=0$ *only.*

### **Theorem 3**

*Suppose that* *s*(*x*) *be the twelfth degree spline defined in section* “Description of the spline (existence and uniqueness)” *and* $f\in C^{13}[0,1]$ *, then for any* $x\in [0,1]$ *we have*

6 $$\begin{aligned} \left| s(x)-f(x)\right| \le \frac{h^{12}}{12!}\left\| f^{(13)}\right\| . \end{aligned}$$

### *Proof*

Since $s'(x)$ is the Hermite interpolation polynomial of degree 11 matching $f^{(j)},\,\,(j=1,2,\ldots ,6)$ at $x=x_{i}$ and $x_{i+1}$. So, by using (2) for $x\in [x_{i},x_{i+1}]$ (with $\; m=6,\,g=f',\;\text {and}\; p_{1}=s'$), we have (see Clarleft et al. 1967)

$$\begin{aligned} \left| s^{(r+1)}(x)-f^{(r+1)}(x)\right| \le \frac{h^{r}[(x-x_i)(x_{i+1}-x)]^{6-r}}{r!(12-2r)!}\left\| f^{(12)}\right\| , \quad r=0(1)6. \end{aligned}$$

Because $x_i\le x\le x_{i+1}$ so that $(x-x_i)(x_{i+1}-x)\le h^2$ and hence

$$\begin{aligned} \left| s^{(r+1)}(x)-f^{(r+1)}(x)\right| \le \frac{h^{12-r}}{r!(12-2r)!}\left\| f^{(12)}\right\| , \quad r=0(1)6. \end{aligned}$$

Then for any $x\in [0,1]$ and $r=0$ this becomes

$$\begin{aligned} \left| s'(x)-f'(x)\right| \le \frac{h^{12}}{(4^{6})(12!)}\left\| f^{(12)}\right\| . \end{aligned}$$

Integrating over [0, *x*] and using $s(0)=f(0)$, the last equation becomes

$$\begin{aligned} \left| s(x)-f(x)\right| \le \frac{h^{12}}{12!}\left\| f^{(13)}\right\| . \end{aligned}$$

Thus we have proved the theorem. $\square$

### *Remark 1*

The inequality (6) provides a correction of inequalities (5), (10), (15.4), (20), and (25) in El Tarazi and Karaballi (1990).

## Algorithms

We have to use following steps for solving a problem:

*Step 1*: Note that the above formulation and analysis was done in [0, 1]. However, this does not constitute a serious restriction since the same techniques can be carried out for the general interval [*a*, *b*]. This is achieved from [*a*, *b*] to [0, 1] using the linear transformation
  7 $$\begin{aligned} x&=\frac{1}{b-a}t-\frac{a}{b-a} \end{aligned}$$
*Step 2*: Use (3) to compute $s_{i}$, $(i=0(1)n)$.
*Step 3*: Use (2) to compute *s*(*x*) at *n* equally spaced points in $[x_{i},x_{i+1}],\;(i=1(1)n)$.

## Illustrations

In this section, we illustrate the numerical technique discussed in the previous section by the following problems, in order to illustrate the comparative performance of the proposed spline method over other existing spline methods. All computations are performed using MATLAB 12b.

### *Example 1*

Consider the following Logarithmic Function (El Tarazi and Karaballi 1990; Rathod et al. 2010):

8 $$\begin{aligned} f(x)=\int _{1}^{x}\frac{dt}{1+t},\quad x\in [1,5]. \end{aligned}$$

**Fig. 1:**
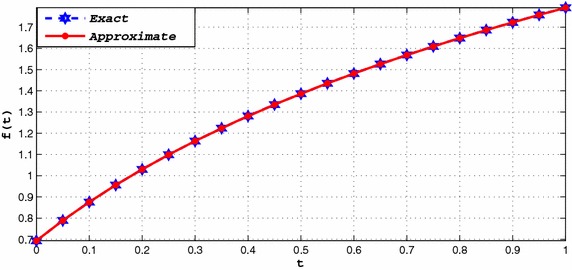
Exact and approximate solutions of Example 1 with $h=1/20$

The numerical solutions using twelfth degree spline are represented in Table 1, in case of $h=0.1$. In Table 2, maximum errors are reported corresponding to the present spline method and the spline method in El Tarazi and Karaballi (1990) for various values of *h*. Tables 3 and 4 show the comparison of the proposed spline method with the standard cubic splines (natural, clamped and a not a knot) and Subbotin cubic spline method developed in Rathod et al. (2010). Also, the exact and the numerical solutions are plotted in Fig. 1 for the step size $h=1/20$. It has been observed that our method is more efficient.

**Table 1:** The numerical solution and exact solution of Example 1

*x*	Exact solution	Approximation solution	Absolute error
0.0	0	0	0
0.1	0.182321556793955	0.182321556793934	2.073341498487480e−014
0.2	0.336472236621213	0.336472236621190	2.303712776097200e−014
0.3	0.470003629245736	0.470003629245712	2.353672812205332e−014
0.4	0.587786664902119	0.587786664902095	2.364775042451583e−014
0.5	0.693147180559945	0.693147180559922	2.364775042451583e−014
0.6	0.788457360364270	0.788457360364247	2.375877272697835e−014
0.7	0.875468737353900	0.875468737353876	2.353672812205332e−014
0.8	0.955511445027436	0.955511445027413	2.375877272697835e−014
0.9	1.029619417181158	1.029619417181135	2.353672812205332e−014
1.0	1.098612288668110	1.098612288668086	2.353672812205332e−014

**Table 2:** Maximum absolute errors in solution Example 1

*h*	Our method	Spline of degree 8 (El Tarazi and Karaballi 1990)	Spline of degree 10 (El Tarazi and Karaballi 1990)
1 / 5	6.3392e−011	2.5e−008	4.5e−010
1 / 10	2.3537e−014	2.3e−010	1.3e−012
1 / 15	2.2204e−016	1.2e−011	3.2e−014
1 / 20	2.2204e−016	1.4e−012	2.7e−015
1 / 25	0	2.5e−013	6.0e−016
1 / 30	2.2204e−016	6.0e−014	5.7e−016

**Table 3:** Numerical results for Example 1

*x*	Cubic Subbotin spline (Rathod et al. 2010)	Natural spline	Our spline (degree 12)	Exact
1.08	0.03922071396027	0.03922071396027	0.039220713153281	0.039220713153281
1.16	0.07696104319944	0.07695999495151	0.076961041136128	0.076961041136128
1.24	0.11332868821323	0.11332791689560	0.113328685307003	0.113328685307003
1.32	0.14842000880542	0.14841915996469	0.148420005118273	0.148420005118273
1.40	0.18232156111573	0.18232073025801	0.182321556793955	0.182321556793955
1.48	0.21511138448704	0.21511054644682	0.215111379616945	0.215111379616945
1.56	0.24686008326688	0.24685924513945	0.246860077931526	0.246860077931526
1.64	0.27763174233409	0.27763090250854	0.277631736598280	0.277631736598280
1.72	0.30748470582867	0.30748386497839	0.307484699747961	0.307484699747961
1.80	0.33647224300070	0.33647140114729	0.336472236621213	0.336472236621213
1.88	0.36464312022710	0.36464227753464	0.364643113587909	0.364643113587909
1.96	0.39204209464187	0.39204125120987	0.392042087776024	0.392042087776024
2.04	0.41871034192254	0.41870949784609	0.418710334858185	0.418710334858185
2.12	0.44468582850026	0.44468498385764	0.444685821261446	0.444685821261446
2.20	0.47000363663837	0.47000279149742	0.470003629245736	0.470003629245736
2.28	0.49469624936477	0.49469540378362	0.494696241836107	0.494696241836107
2.36	0.51879380106450	0.51879295509338	0.518793793415168	0.518793793415168
2.44	0.54232429858202	0.54232345226443	0.542324290825362	0.542324290825362
2.52	0.56531381690245	0.56531297027618	0.565313809050060	0.565313809050060
2.60	0.58778667284010	0.58778582593813	0.587786664902119	0.587786664902119
2.68	0.60976557963560	0.60976473248676	0.609765571620894	0.609765571620894
2.76	0.63127178492549	0.63127093755506	0.631271776841858	0.631271776841858
2.84	0.65232519418539	0.65232434661562	0.652325186039690	0.652325186039690
2.92	0.67294448144414	0.67294363369464	0.672944473242426	0.672944473242426
3.00	0.69314718881230	0.69314634090043	0.693147180559945	0.693147180559945
3.08	0.71294981615437	0.71294896809550	0.712949807856125	0.712949807856125
3.16	0.73236790205313	0.73236705386091	0.732367893713227	0.732367893713227
3.24	0.75141609706171	0.75141524874832	0.751416088683921	0.751416088683921
3.32	0.77010823010838	0.77010738168467	0.770108221696074	0.770108221696074
3.40	0.78845736880808	0.78845652028376	0.788457360364270	0.788457360364270
3.48	0.80647587433955	0.80647502572333	0.806475865866949	0.806475865866949
3.56	0.82417545146531	0.82417460276501	0.824175442966349	0.824175442966349
3.64	0.84156719420135	0.84156634542400	0.841567185678219	0.841567185678219
3.72	0.85866162758285	0.85866077873478	0.858661619037519	0.858661619037519
3.80	0.87546874591965	0.87546789700658	0.875468737353900	0.875468737353900
3.88	0.89199804788966	0.89199719891678	0.891998039305110	0.891998039305110
3.96	0.90825856877877	0.90825771975076	0.908258560176891	0.908258560176891
4.04	0.92425891014122	0.92425806106235	0.924258901523332	0.924258901523332
4.12	0.94000726712415	0.94000641799821	0.940007258491471	0.940007258491471
4.20	0.95551145367381	0.95551060450466	0.955511445027437	0.955511445027436
4.28	0.97077892581729	0.97077807660683	0.970778917158225	0.970778917158225
4.36	0.98581680319361	0.98581595394963	0.985816794522765	0.985816794522765
4.44	1.00063188898968	1.00063103969670	1.000631880307906	1.000631880307906
4.52	1.01523068842100	1.01522983914930	1.015230679729059	1.015230679729059
4.60	1.02961942588257	1.02961857638119	1.029619417181158	1.029619417181158
4.68	1.04380406088334	1.04380321209931	1.043804052173115	1.043804052173115
4.76	1.05779030286630	1.05778945127489	1.057790294147855	1.057790294147855
4.84	1.07158362500631	1.07158278377077	1.071583616280190	1.071583616280190
4.92	1.08518927706926	1.08518839707176	1.085189268335969	1.085189268335969
5.00	1.09861229740415	1.09861156196633	1.098612288668110	1.098612288668110

**Table 4:** Numerical results for Example 1

*x*	Clamped spline	Not a knot spline	Our spline (degree 12)	Exact
1.08	0.03922071396027	0.03922071396027	0.039220713153281	0.039220713153281
1.16	0.07696103950333	0.07696102915780	0.076961041136128	0.076961041136128
1.24	0.11332868156060	0.11332867398715	0.113328685307003	0.113328685307003
1.32	0.14841999962514	0.14841999130891	0.148420005118273	0.148420005118273
1.40	0.18232154982349	0.18232154170628	0.182321556793955	0.182321556793955
1.48	0.21511137139673	0.21511136322620	0.215111379616945	0.215111379616945
1.56	0.24686006864661	0.24686006049037	0.246860077931526	0.246860077931526
1.64	0.27763172640228	0.27763171824221	0.277631736598280	0.277631736598280
1.72	0.30748468876854	0.30748468060950	0.307484699747961	0.307484699747961
1.80	0.33647222496520	0.33647221680588	0.336472236621213	0.336472236621213
1.88	0.36464310134512	0.36464309318587	0.364643113587909	0.364643113587909
1.96	0.39204207502234	0.39204206686307	0.392042087776024	0.392042087776024
2.04	0.41871032165802	0.41871031349876	0.418710334858185	0.418710334858185
2.12	0.44468580766972	0.44468579951046	0.444685821261446	0.444685821261446
2.20	0.47000361530946	0.47000360715019	0.470003629245736	0.470003629245736
2.28	0.49469622759567	0.49469621943641	0.494696241836107	0.494696241836107
2.36	0.51879377890542	0.51879377074616	0.518793793415168	0.518793793415168
2.44	0.54232427607647	0.54232426791721	0.542324290825362	0.542324290825362
2.52	0.56531379408823	0.56531378592897	0.565313809050060	0.565313809050060
2.60	0.58778664975018	0.58778664159092	0.587786664902119	0.587786664902119
2.68	0.60976555629880	0.60976554813954	0.609765571620894	0.609765571620894
2.76	0.63127176136711	0.63127175320785	0.631271776841858	0.631271776841858
2.84	0.65232517042767	0.65232516226841	0.652325186039690	0.652325186039690
2.92	0.67294445750669	0.67294444934743	0.672944473242426	0.672944473242426
3.00	0.69314716471248	0.69314715655322	0.693147180559945	0.693147180559945
3.08	0.71294979190754	0.71294978374828	0.712949807856125	0.712949807856125
3.16	0.73236787767296	0.73236786951370	0.732367893713227	0.732367893713227
3.24	0.75141607256037	0.75141606440110	0.751416088683921	0.751416088683921
3.32	0.77010820549672	0.77010819733746	0.770108221696074	0.770108221696074
3.40	0.78845734409581	0.78845733593655	0.788457360364270	0.788457360364270
3.48	0.80647584953538	0.80647584137612	0.806475865866949	0.806475865866949
3.56	0.82417542657706	0.82417541841780	0.824175442966349	0.824175442966349
3.64	0.84156716923605	0.84156716107678	0.841567185678219	0.841567185678219
3.72	0.85866160254683	0.85866159438757	0.858661619037519	0.858661619037519
3.80	0.87546872081863	0.87546871265937	0.875468737353900	0.875468737353900
3.88	0.89199802272882	0.89199801456956	0.891998039305110	0.891998039305110
3.96	0.90825854356281	0.90825853540355	0.908258560176891	0.908258560176891
4.04	0.92425888487438	0.92425887671512	0.924258901523332	0.924258901523332
4.12	0.94000724181031	0.94000723365105	0.940007258491471	0.940007258491471
4.20	0.95551142831649	0.95551142015722	0.955511445027437	0.955511445027436
4.28	0.97077890041969	0.97077889226042	0.970778917158225	0.970778917158225
4.36	0.98581677775865	0.98581676959939	0.985816794522765	0.985816794522765
4.44	1.00063186352005	1.00063185536077	1.000631880307906	1.000631880307906
4.52	1.01523066291915	1.01523065475995	1.015230679729059	1.015230679729059
4.60	1.02961940035073	1.02961939219125	1.029619417181158	1.029619417181158
4.68	1.04380403532359	1.04380402716516	1.043804052173115	1.043804052173115
4.76	1.05779027728051	1.05779026911814	1.057790294147855	1.057790294147855
4.84	1.07158359939627	1.07158359124860	1.071583616280190	1.071583616280190
4.92	1.08518925143643	1.08518924323388	1.085189268335969	1.085189268335969
5.00	1.09861227175446	1.09861226375673	1.098612288668110	1.098612288668110

### *Example 2*

Consider the following function (Phythian and Williams 1986):

9 $$\begin{aligned} g(u)=u^4+1,\quad u\in [1,2]. \end{aligned}$$

**Fig. 2:**
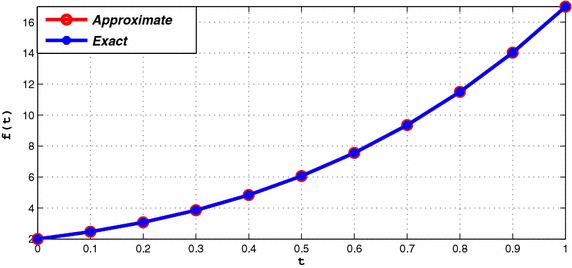
Exact and approximate solutions of Example 2 with $h=1/40$

The numerical solutions using twelfth degree spline are represented in Table 5, for $h=0.1$. The maximum absolute errors are tabulated in Table 6 for various values of *h* and compared with Anwar and El-Tarazi (1989). Figure 2 illustrates the comparison of numerical solution and analytical solution values at $h=1/40$. The proposed numerical solution gives almost overlapping behavior with the corresponding exact solution values.

**Table 5:** The numerical solution and exact solution of Example 2

*u*	Exact solution	Approximation solution	Absolute error
0.0	2	2	0
0.1	2.464100000000001	2.464100000000000	4.440892098500626E−16
0.2	3.073600000000000	3.073600000000000	4.440892098500626E−16
0.3	3.856100000000001	3.856100000000001	0
0.4	4.841599999999999	4.841600000000001	1.776356839400251E−15
0.5	6.062500000000000	6.062500000000000	0
0.6	7.553600000000001	7.553600000000000	8.881784197001252E−16
0.7	9.352099999999998	9.352100000000000	1.776356839400251E−15
0.8	11.497600000000002	11.497600000000000	1.776356839400251E−15
0.9	14.032099999999998	14.032100000000000	1.776356839400251E−15
1.0	17	17	0

**Table 6:** The maximum absolute errors for Example 2

Step size *h*	Our method	Method in Anwar and El-Tarazi (1989)
0.1	0	3.3e−005
0.05	0	2.1e−006
0.025	0	1.3e−007
0.02	0	5.3e−008
0.0125	3.5527e−015	8.1e−009
0.01	0	3.3e−009

### *Example 3*

Consider the Indefinite integral of Runge Function (Rathod et al. 2010):

10 $$\begin{aligned} f(x)=\int _{-1}^{x}\frac{dt}{1+25t^2},\quad x\in [-1,1]. \end{aligned}$$

Table 7 demonstrates the comparison of the proposed spline method with the Subbotin cubic spline method developed in Rathod et al. (2010).

**Table 7:** Numerical results for Example 3

*x*	Cubic Subbotin spline (Rathod et al. 2010)	Our spline (degree 12)	Exact
−0.96	0.00159996602042	0.001599965867977	0.001599965867977
−0.92	0.00333302534289	0.003333024742788	0.003333024742788
−0.88	0.00521620933478	0.005216208258150	0.005216208258151
−0.84	0.00726952592022	0.007269524203804	0.007269524203804
−0.80	0.00951662318369	0.009516620655397	0.009516620655397
−0.76	0.01198563460978	0.011985631024242	0.011985631024242
−0.72	0.01471026306531	0.014710258097708	0.014710258097708
−0.68	0.01773118316554	0.017731176373487	0.017731176373487
−0.64	0.02109787092452	0.021097861705018	0.021097861705018
−0.60	0.02487101138281	0.024870998909352	0.024870998909352
−0.56	0.02912569297475	0.029125676114165	0.029125676114165
−0.52	0.03395567758607	0.033955654793668	0.033955654793668
−0.48	0.03947914276296	0.039479111969976	0.039479111969976
−0.44	0.04584642810741	0.045846386655399	0.045846386655399
−0.40	0.05325046504333	0.053250409830185	0.053250409830185
−0.36	0.06194066062908	0.061940588908491	0.061940588908491
−0.32	0.07224083917045	0.072240751098736	0.072240751098736
−0.28	0.08457087983609	0.084570785226588	0.084570785226588
−0.24	0.09946860939441	0.099468543269364	0.099468543269364
−0.20	0.11760046918276	0.117600520709514	0.117600520709514
−0.16	0.13973162302703	0.139731964944293	0.139731964944293
−0.12	0.16659542193349	0.166596253334887	0.166596253334886
−0.08	0.19857766701988	0.198578877966528	0.198578877966530
−0.04	0.23520031780477	0.235201041419030	0.235201041419027
0.00	0.27468081092706	0.274680153389003	0.274680153389003
0.04	0.31416062591739	0.314159265358976	0.314159265358979
0.08	0.35078230627314	0.350781428811479	0.350781428811476
0.12	0.38276433473857	0.382764053443120	0.382764053443120
0.16	0.40962833358333	0.409628341833713	0.409628341833714
0.20	0.43175968738734	0.431759786068493	0.431759786068493
0.24	0.44989165576688	0.449891763508642	0.449891763508642
0.28	0.46478942923129	0.464789521551418	0.464789521551418
0.32	0.47711948282318	0.477119555679270	0.477119555679270
0.36	0.48741966211928	0.487419717869515	0.487419717869515
0.40	0.49610985468976	0.496109896947821	0.496109896947821
0.44	0.50351388804248	0.503513920122607	0.503513920122607
0.48	0.50988117027174	0.509881194808030	0.509881194808030
0.52	0.51540463300912	0.515404651984338	0.515404651984339
0.54	0.52023461579352	0.520234630663842	0.520234630663842
0.60	0.52448929604318	0.524489307868654	0.524489307868654
0.64	0.52826243552178	0.528262445072988	0.528262445072988
0.68	0.53162912256560	0.531629130404519	0.531629130404519
0.72	0.53465004214157	0.534650048680298	0.534650048680298
0.76	0.53737467021086	0.537374675753765	0.537374675753765
0.80	0.53984368134898	0.539843686122609	0.539843686122610
0.84	0.54209077839986	0.542090782574202	0.542090782574202
0.88	0.54414409481617	0.544144098519856	0.544144098519856
0.92	0.54602727870406	0.546027282035218	0.546027282035218
0.96	0.54776033787596	0.547760340910029	0.547760340910029
1.00	0.54936030383282	0.549360306778006	0.549360306778006

### Example 4

Finally, we consider the Normal Distribution (Rathod et al. 2010):

11 $$\begin{aligned} f(x)=\int _{0}^{x}\frac{e^{-\frac{t^2}{2}}}{\sqrt{2\pi }}dt,\quad x\in [0,4]. \end{aligned}$$

The numerical results are tabulated in Table 8. Also comparison is made with the existing method in Rathod et al. (2010).

It is clear from the Tables 1, 2, 3, 4, 5, 6, 7 and 8 that our methods are better than the other existing methods. The results of our methods are better than those has lower order (Anwar and El-Tarazi 1989; El Tarazi and Karaballi 1990; Rathod et al. 2010).

**Table 8:** Numerical results for Example 4

*x*	Cubic Subbotin spline (Rathod et al. 2010)	Our spline (degree 12)	Exact
0.08	0.03188137343786	0.03188137201399	0.03188137201399
0.16	0.06355946719297	0.06355946289143	0.06355946289143
0.24	0.09483487811231	0.09483487169780	0.09483487169780
0.32	0.12551584316831	0.12551583472332	0.12551583472332
0.40	0.15542175168553	0.15542174161032	0.15542174161032
0.48	0.18438631483954	0.18438630348378	0.18438630348378
0.56	0.21226029337979	0.21226028115097	0.21226028115097
0.64	0.23891371300311	0.23891370030714	0.23891370030714
0.72	0.26423751498017	0.26423750222075	0.26423750222075
0.80	0.28814461385800	0.28814460141660	0.28814460141660
0.88	0.31057035699849	0.31057034522329	0.31057034522329
0.96	0.33147240333869	0.33147239253316	0.33147239253316
1.04	0.35083005925386	0.35083004966900	0.35083004966902
1.12	0.36864312712815	0.36864311895727	0.36864311895727
1.20	0.38493033640194	0.38493032977830	0.38493032977829
1.28	0.39972743704822	0.39972743204556	0.39972743204556
1.36	0.41308504141739	0.41308503805292	0.41308503805292
1.44	0.42506630222622	0.42506630046567	0.42506630046567
1.52	0.43574451241669	0.43574451218106	0.43574451218106
1.60	0.44520070712697	0.44520070830044	0.44520070830044
1.68	0.45352133969787	0.45352134213630	0.45352134213628
1.76	0.46079609317294	0.46079609671252	0.46079609671252
1.84	0.46711587687507	0.46711588134084	0.46711588134084
1.92	0.47257104508264	0.47257105029616	0.47257105029616
2.00	0.47724986226558	0.47724986805180	0.47724986805182
2.08	0.48123722737203	0.48123723356506	0.48123723356506
2.16	0.48461365876857	0.48461366521607	0.48461366521607
2.24	0.48745453199754	0.48745453856405	0.48745453856405
2.32	0.48982955476235	0.48982956133128	0.48982956133128
2.40	0.49180245760085	0.49180246407540	0.49180246407540
2.48	0.49343087456130	0.49343088086445	0.49343088086445
2.56	0.49476638576280	0.49476639183644	0.49476639183644
2.64	0.49585469283547	0.49585469863896	0.49585469863896
2.72	0.49673589867582	0.49673590418410	0.49673590418411
2.80	0.49744486446812	0.49744486966957	0.49744486966957
2.88	0.49801161925099	0.49801162414511	0.49801162414511
2.96	0.49909574075267	0.49846180478826	0.49846180478826
3.04	0.49931285825549	0.49881710925690	0.49881710925690
3.12	0.49948096097656	0.49909574480018	0.49909574480018
3.20	0.49961028423860	0.49931286206208	0.49931286206208
3.28	0.49970913967489	0.49948096456679	0.49948096456679
3.36	0.49978422351271	0.49961028763743	0.49961028763742
3.44	0.49984088844420	0.49970914290671	0.49970914290671
3.52	0.49988338016042	0.49978422660071	0.49978422660071
3.60	0.49991504054429	0.49984089140984	0.49984089140984
3.68	0.49993848013798	0.49988338302318	0.49988338302318
3.76	0.49993848013798	0.49991504332150	0.49991504332150
3.84	0.49993848013798	0.49993848284482	0.49993848284482
3.92	0.49995572286615	0.49995572551569	0.49995572551569
4.00	0.49996832612598	0.49996832875817	0.49996832875817

## Conclusion

In this paper, the existence and uniqueness of a twelfth degree spline are derived and in which we have obtained a direct simple formula. This formula is agree with those obtained in Clarleft et al. (1967), El Tarazi and Karaballi (1990), Phythian and Williams (1986), where a different approach was used. Moreover, the performance of the proposed twelfth degree spline with the even degree splines (El Tarazi and Karaballi 1990), direct cubic spline (Anwar and El-Tarazi 1989), standard cubic splines (natural, clamped and a not a knot) and Subbotin cubic spline (Rathod et al. 2010; Rathod et al. 2010). For which, error estimates and numerical examples are presented. On the basis of the examples, the proposed method yields much better results than the other methods. Also, a mistake is corrected in the literature that occurred in error bounds.
